# A mixed-methods longitudinal observational study exploring physical activity during pregnancy in women with pre-existing diabetes, support needs and associations with diabetes management: a study protocol

**DOI:** 10.1136/bmjopen-2026-118879

**Published:** 2026-06-10

**Authors:** Holly Mei Jones, Robert Andrews, Emma J Cockcroft, Isy F Douek, Richard M Pulsford

**Affiliations:** 1 Public Health and Sport Sciences, University of Exeter, Exeter, UK; 2 Department of Diabetes, Taunton and Somerset Hospital, Taunton, UK; 3 NIHR Exeter, Biomedical Research Centre, Exeter, UK; 4 Health and Community Sciences, University of Exeter, Exeter, UK

**Keywords:** Exercise, Pregnancy, Diabetes Mellitus, Type 1, Diabetes Mellitus, Type 2

## Abstract

**Introduction:**

Pregnancy in women with pre-existing type 1 or type 2 diabetes (T1D, T2D) is associated with increased risk of complications, largely driven by maternal glucose control. Hormonal changes during pregnancy make glucose management more challenging. Physical activity (PA) may improve glucose control and reduce complications; however, little is known about PA patterns in this population and no pregnancy-specific PA guidance exists for women with pre-existing diabetes. Understanding the behaviours and experiences of both pregnant women and the healthcare professionals (HCPs) who support them is needed to inform evidence-based guidance.

**Methods and analysis:**

This mixed-methods study comprises three sub-studies. The first will recruit 175 pregnant women (75 with T1D and 100 with T2D) who will complete three 7-day monitoring periods, one per trimester. PA will be assessed using wrist-worn accelerometers and exercise diaries, dietary intake via remote food photography, and corresponding continuous glucose monitor and diabetes-related well-being data will be collected.

The second involves a subsample of ~16 women participating in focus groups to explore experiences of being physically active during pregnancy.

The third invites ~100 HCPs involved in diabetes in pregnancy care to complete an online survey, ~10 HCPS will take part in an optional interview about their experiences of providing PA guidance.

The primary outcome is the change in PA across pregnancy. Secondary outcomes include associations between PA, glucose metrics, diet and diabetes-related well-being, and qualitative themes relating to experiences of women and HCP. Quantitative data will be analysed using multilevel modelling and regression analysis, and qualitative data using reflexive thematic analysis.

**Ethics and dissemination:**

Ethical approval was granted by the East Midlands Nottingham 1 Research Ethics Committee (25/EM/0190) and University of Exeter Public Health and Sport Sciences ethics committee. Findings will be disseminated through peer-reviewed publications and conference presentations.

**STRENGTHS AND LIMITATIONS OF THIS STUDY**

- The use of accelerometers allows precise and unbiased measurement of physical activity (PA), providing detailed insight into habitual and temporal activity patterns.
- Longitudinal study design, involving tracking participants across three trimesters, allows us to explore the changes over time, rather than relying on a single time point.
- Mixed methods approach using quantitative and qualitative data from women and healthcare professionals provides a rich and holistic understanding of PA behaviours in pregnant women with pre-existing type 1 or type 2 diabetes.
- Recruitment may be skewed towards the southwest region, specific ethnic groups and those more engaged with care or an interest in PA.

## Introduction

 Pregnancy is an important transitional period involving physiological and behavioural changes that affect physical health and mental well-being.^1–3^ Women with pre-existing type 1 or type 2 diabetes (T1D, T2D) face additional challenges related to complexities of diabetes management. The prevalence of pre-existing diabetes in pregnancy is rising, largely due to increasing rates of T2D.^4 5^ In 2024, there were 2240 recorded pregnancies in women with T1D, and 3520 in women with T2D.^6^ However, this is an underestimate of the true prevalence, as early pregnancy loss is more common in women with pre-existing diabetes^7^ and is less well documented.

Pregnancies affected by pre-existing diabetes are associated with an increased risk of complications such as miscarriage, preterm delivery and macrosomia.^7–10^ These risks are primarily driven by maternal hyperglycaemia and glycaemia variability. Achieving optimal glucose control during pregnancy is essential, with recommended targets <5.3 mmol/L fasting and <7.8 mmol/L postprandial,^11^ compared with <7 mmol/L and <9 mmol/L outside pregnancy.

Achieving narrower glucose control is challenging due to pregnancy-related hormonal changes which influence insulin sensitivity and increase hepatic glucose production to support fetal growth.^12–14^ With advancing gestation, insulin resistance rises, often requiring threefold to fourfold increase in insulin.^13^ These changes make glucose control unpredictable and challenging, increasing both the risk of hypoglycaemia and hyperglycaemia.^13^ Despite advances in diabetes technologies, including continuous glucose monitor (CGM) and hybrid closed-loop systems, maternal and fetal outcomes remain poorer than in pregnancies without diabetes.^8^

These physiological demands combine with mental pressures.^15^ Women have described the relentless demands to monitor glucose, adjust insulin and adhere to dietary guidance,^16^ contributing to anxiety and self-blame.^17^ Supporting women requires approaches that address both physical and mental demands during pregnancy.

Regular physical activity (PA), including structured exercise, may benefit both physical health and mental well-being. During uncomplicated pregnancies, PA reduces the risk of adverse outcomes and improves cardiometabolic health and mood.^18^ Among adults living with diabetes outside pregnancy, PA improves glucose control, reduces insulin needs and improves cardiometabolic outcomes.^19–21^ However, many women reduce PA and structured exercise during uncomplicated pregnancies, and only 1 in 10 meet the UK Chief Medical Officer’s guidelines of 150 min of moderate-intensity PA per week.^22^

Research exploring PA patterns during pregnancy in women with pre-existing diabetes is sparse, with few longitudinal studies, limiting insight into PA across gestation.^23–25^ Most existing research relies on self-report measures of PA,^25^ which are prone to recall error and bias^26^ and may misrepresent habitual activity levels and obscure associations with diabetes and pregnancy outcomes. Although device-based measures of PA, including wearable sensors such as accelerometers, offer more precise and unbiased data on PA behaviours, their application in this population is rare.^27–29^

To date, only one published study has combined device-measured PA with modern diabetes technology in this population. In 10 pregnant women with T1D, an increased risk of hypoglycaemia was observed following a single structured exercise bout, compared with during free-living. This provides limited insight into free-living PA–glucose relationships in women with T1D. No such evidence exists for women with T2D.^27^

Qualitative research exploring the lived experiences during pregnancy of women with pre-existing diabetes is limited, and existing studies have largely focused on experiences of diabetes management rather than PA specifically.^30 31^ Notably, no studies have explored the approaches of healthcare professionals (HCPs) towards PA guidance, the barriers they face or what support they need. These perspectives are essential for developing safe, acceptable and context-specific PA guidance.

Taken together, existing research provides limited understanding of how active women with pre-existing diabetes are during pregnancy, how PA changes across gestation and how PA relates to glucose control or mental well-being. Evidence is similarly lacking on the lived experiences of women, their support needs and the perspectives of HCPs responsible for advising on PA. This absence of evidence makes it difficult to provide evidence-based, specific and context-appropriate PA guidance for women with pre-existing diabetes.

This proposed mixed methods study aims to address these key gaps by combining contemporaneous longitudinal assessment of habitual PA and glucose levels in pregnant women with pre-existing diabetes, with qualitative exploration of the lived experiences and support preferences of women, alongside a survey and qualitative exploration of experiences and support preferences of HCPs. Findings will inform evidence-based guidance to support HCPs in promoting safe and beneficial PA in this population.

### Study aims

The primary aim of the study is to undertake a mixed-methods longitudinal observational study to assess changes in PA throughout pregnancy in women with pre-existing T1D or T2D, to understand how PA changes throughout pregnancy and when in pregnancy PA guidance may be most beneficial.

This aim will be met through the following objectives:

1. Measure changes in PA across pregnancy.
2. Examine associations between PA, glucose control and dietary intake.
3. Explore associations between PA and diabetes-related emotional well-being.
4. Explore the experience of pregnant women, barriers, facilitators and support needs related to PA.
5. Explore the experiences of HCPs in promoting PA, including confidence, perceived barriers and resource needs.

## Methods and analysis

### Study design and overview

This protocol uses a mixed-methods design comprising three components: (1) a longitudinal observational cohort study examining changes in PA and glucose metrics in pregnant women with pre-existing diabetes; (2) focus groups exploring the lived experiences of women; and (3) a national survey and interviews with HCPs to examine practices and resource needs for PA promotion. Together, these components provide complementary data to inform evidence-based recommendations.

The longitudinal observational cohort study will be conducted across NHS sites in the South West of England. Pregnant women with T1D or T2D will complete three measurement periods aligned with pregnancy trimesters. During each period, PA and exercise will be assessed over 7 days using wrist-worn accelerometers and exercise diaries, diet will be captured using remote food photographs, and CGM data for the corresponding period will be shared with the research team. Participants will complete a demographic questionnaire at baseline, and a diabetes distress questionnaire at the end of each measurement period.

Participants enrolled into this observational study will have the option to attend a focus group to discuss experiences and perceived barriers or enablers to PA or exercise during pregnancy, and what support they feel would be most useful.

Some sites will act as participant identification centres (PICs). PIC sites will identify eligible participants during routine clinical care and, with consent, will share the contact details of participants securely with the University of Exeter research team. The research team will then undertake all study procedures remotely, including provision of participant information, consent, posting out accelerometers and coordination of data collection. This approach reduces the research burden on National Health Service (NHS) sites and enhances efficiency of recruitment while enabling wider geographical reach and improving the diversity of the study population.

A parallel study will examine the experiences of HCPs promoting PA in this population. HCPs involved in the support or care of these women will be invited to complete an online survey and attend a semi-structured interview. The survey involves Likert scale responses and open-ended questions to assess frequency and confidence in HCPs providing PA advice. Semi-structured interviews will be completed on an online video conferencing service, with questions to assess barriers and facilitators to PA promotion, knowledge gaps and training needs.

**Figure 1:**
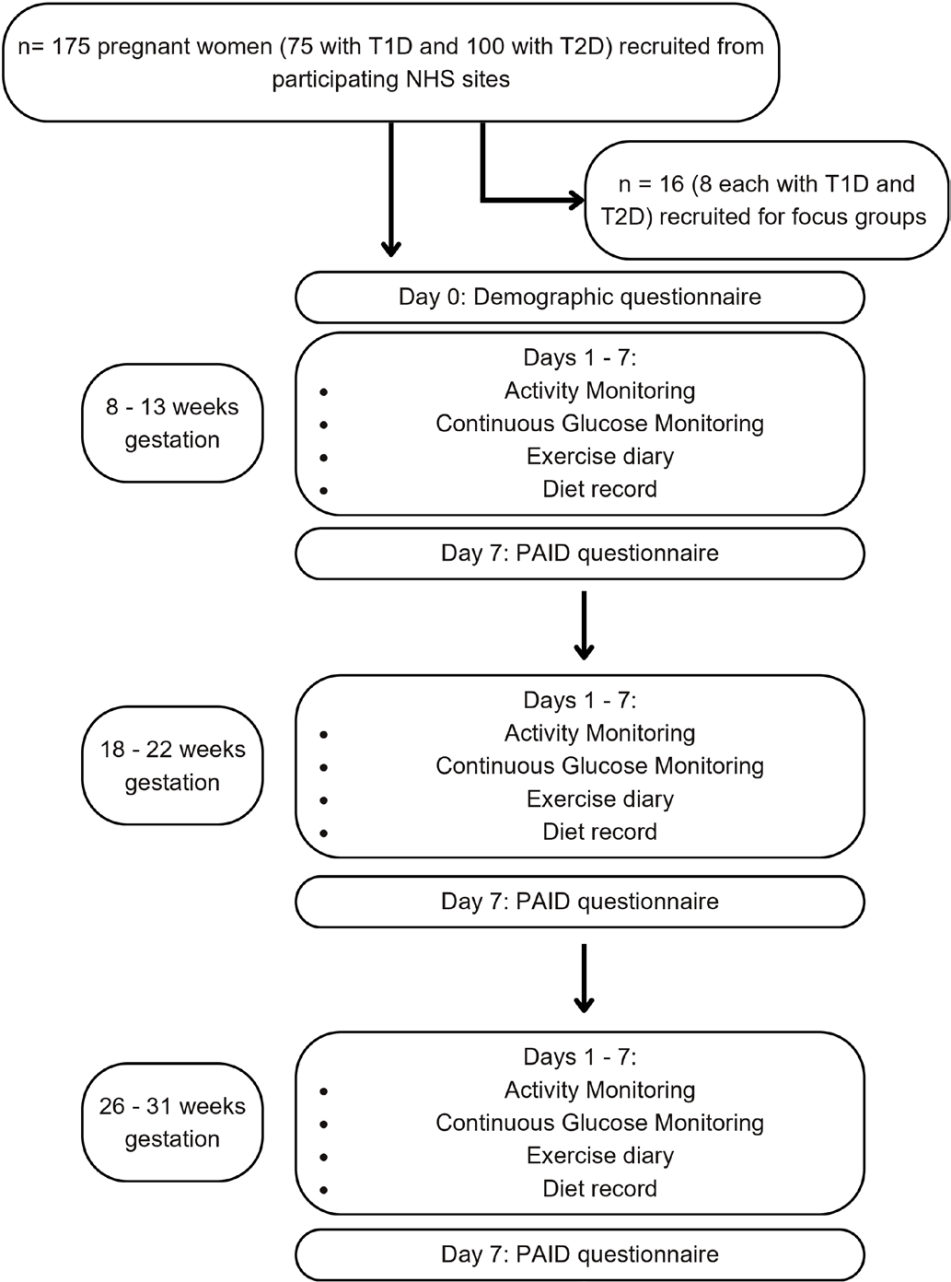
Protocol for objectives 1–4. NHS, National Health Service; PAID, problem areas in diabetes; T1D, type 1 diabetes; T2D, type 2 diabetes.

**Figure 2:**
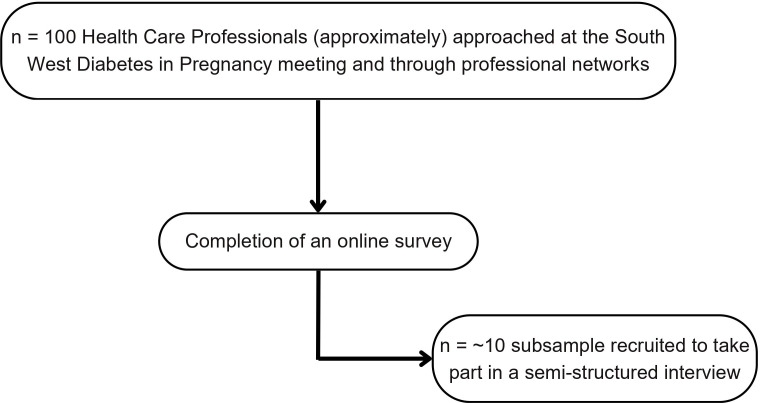
Protocol for objective 5.

An overview of the protocol design is shown in figures 1 and 2.

### Study setting and recruitment plan

Pregnant women (>18 years) with T1D or T2D will be recruited between 8 and 12 weeks’ gestation from NHS diabetes clinics in the South West of England between March 2026 and September 2027. Recruitment may be skewed towards the South West region, specific ethnic groups and those engaged with care or an interest in PA. To mitigate this, we will recruit across multiple NHS sites, including PICs to widen the geographic reach, which serve socioeconomically and ethnically diverse communities. In addition to this, we will closely monitor recruitment demographics throughout the study to ensure the inclusion of individuals from ethnic minority backgrounds.

The target sample to inform our primary analyses is 175 participants (75 T1D and 100 T2D), with a sub-sample of approximately 16 of these participants recruited to take part in focus groups. Participants will be recruited through direct approach by research nurses at routine diabetes clinic visits at participating NHS sites, supported by posters in waiting rooms. Interested participants will be provided with a participant information sheet, and if agreeable, will provide informed consent via an electronic consent form. Participants will have the option to opt-in to be contacted about a focus group.

HCPs will be recruited via a convenience sample from the South West Diabetes in Pregnancy meeting in May 2025 and 2026, attended by ~100 HCPs involved in the care of women with pre-existing diabetes, and through professional networks.

### Sample size

We anticipate that the changes in PA across trimesters of pregnancy in women with diabetes will be at least as large as those seen in women without diabetes. Therefore, this study has been powered to detect a decrease in 6 min of moderate-to-vigorous physical activity (MVPA) per day between trimesters based on the findings of two studies of women without diabetes (MVPA decreased by 8 min/day (SD±14, n=87 women) from trimesters 2 and 3; MVPA decreased by 4.5 min/day (SD±14, n=76 obese women)).^32 33^ The magnitude of change in PA in women with pre-existing diabetes during pregnancy is unknown; therefore, the sample size has been calculated based on healthy and obese pregnant populations.

We have calculated that the study will need to recruit n=175 pregnant women with diabetes (n=75 with T1D and n=100 with T2D) in order to have 80% power of detecting a statistically significant (p<0.05) decrease in daily minutes of MVPA, when the true decrease is ≥6 min/day (SD±14, D=0.4) while accounting for the small proportion of women who are not seen in clinic before 10–12 weeks of pregnancy (approximately 15% of women with T1D and 35% of women with T2D), miscarriage rates (5%) and the potential for a small clustering effect across the participating sites (ICC=0.01). This sample size was calculated with the aid of G*Power software.^34^

Participants for the focus groups will be recruited from the observational cohort using a purposive sampling framework. While participation in the qualitative component is voluntary, sampling will be guided by predefined characteristics to support diversity in ethnicity and socioeconomic status. Characteristics of interested participants will be monitored, and invitations will be issued to ensure representation across key demographic groups where feasible.

We anticipate recruiting ~16 pregnant women at different stages in pregnancy (8 each with T1D and T2D) for the focus groups or interviews in groups of up to 4. The a priori determination of a precise sample size is uncommon in qualitative research. Instead, sampling will be guided by data adequacy, defined as the point at which the research team has generated sufficient relevant information to address the research question, while considering the parameters of the research and the pragmatic limits of the study duration and design.^35^ Samples in qualitative research are purposive and need to be small enough to support the in-depth case-oriented analysis that is fundamental to this mode of enquiry. Data adequacy will be assessed through ongoing, iterative analysis during data collection. Recruitment will continue until the research team determines that additional focus groups or interviews are no longer generating new, substantively meaningful insights and that the data addresses the research questions.

For the survey and semi-structured interviews with HCPs, we anticipate recruiting ~100 for the survey and 10 HCPs for the interviews. Due to the recruitment method, a predetermined sample size was not feasible, this is in line with other exploratory surveys and will allow us to gain a detailed understanding of the topic. As per the above, in qualitative research, a precise sample size is uncommon.

### Eligibility criteria

Eligible participants for objectives 1–4 will be pregnant women with a diagnosis of T1D or T2D prior to conception.

### Inclusion criteria

- Women between 8 and 20 weeks pregnant (this will allow completion of at least two measures during pregnancy).
- Singleton pregnancy.
- Confirmed diagnosis of T1D or T2D.
- Over 18 years old.
- Willing and able to provide informed consent.
- Receiving care at one of the participating NHS sites.

### Exclusion criteria

- Multiple pregnancy.
- Absolute contraindication to PA according to 2019 Canadian Guidelines for PA during pregnancy; examples include ruptured membranes, preeclampsia and intrauterine growth restriction. The full list is available in the Candan Guideline for Physical Activity throughout Pregnancy.^36^
- No diagnoses of T1D or T2D prior to pregnancy.
- Gestational diabetes.

Rationale: women with gestational diabetes are excluded as it arises during pregnancy (typically diagnosed during late second trimester) and has differing clinical management from T1D and T2D.

Eligible participants for objective five are HCPs who provide care for pregnant women with pre-existing T1D or T2D.

### Inclusion criteria

- HCPs who provide care for pregnant women with pre-existing T1D or T2D.
- Willingness to complete a survey and an optional interview about their experiences and views on promoting PA during pregnancy.

### Exclusion criteria

- HCPs who do not work directly with pregnant women with T1D or T2D.
- Students or trainees who are not fully qualified.

### Outcomes

The primary outcome is the change in daily minutes of MVPA across trimesters of pregnancy. In addition, the study has been designed to examine the following secondary outcomes: (1) the associations between PA and glucose control, (2) the associations between PA and problem areas in diabetes (PAID) questionnaire responses, and (3) experiences of, and barriers and facilitators to being physically active during pregnancy.

This observational study will be reported in line with the Strengthening the Reporting of Observational Studies in Epidemiology (STROBE) checklist, specific to observational studies,^37^ and the qualitative studies will be reported in line with the Consolidated criteria for Reporting Qualitative research (COREQ) checklist, specific to qualitative studies.^38^

### Data collection procedures

Table 1 provides a summary of all study measures. These are described in detail below.

1. Demographic and clinic data

participant demographic information, including age, height, weight, ethnicity, first three digits of postcode and date of diabetes diagnosis, will be collected on enrolment via an electronic participant questionnaire (Microsoft Forms).

**Table 1:** Summary of study measures

Measure	Description	Timepoint/s of evaluation	Measurement method
Participant demographic information	Age, height, weight, ethnicity, first three digits of post code and date of diagnosis	Single assessment on enrolment	Electronic participant questionnaire
PA	Daily minutes if MVPA, total PA and sedentary time	Up to three measures, single measure in each trimester.	Wrist-worn activity monitor, worn for 7 days
Exercise	Timing and type of exercise	Up to three measures, single measure in each trimester.	Exercise diary including 7-day recall and 7-day prospective diary
Sleep	Sleep and waking time over 7 days	Up to three measures, single measure in each trimester.	Included in exercise diary
Meals	Photo taken of meals (one before eating and one when finished) for 7 days.	Up to three measures, single measure in each trimester.	Remote food photography
Glucose and insulin	Consensus metrics of glycaemic variability	Up to three measures, single measure in each trimester.	Continuous glucose monitor and insulin pump data shared by patients with clinic teams.
Diabetes-related emotional well-being	Twenty-item questionnaire to assess the emotional well-being experiences of individuals with diabetes.	Up to three measures, single measure in each trimester.	PAID-20 item questionnaire
Pregnancy outcomes	Number of prior pregnancies, outcome of current pregnancy and birth weight of baby	Recorded routinely throughout and after pregnancy.	Collected routinely in clinics by research nurses

MVPA, moderate-to-vigorous physical activity; PA, physical activity; PAID, problem areas in diabetes.

Clinical data, including pregnancy history, current pregnancy outcomes and birth weight of baby, will be routinely collected by research nurses during standard antenatal and postnatal visits.

These data will support subgroup analyses and adjustment of statistical models for potential confounders.

1. Assessment of habitual PA and exercise

PA will be measured using both device-based and self-reported methods.

Device-measured PA: participants will wear a GENEActiv wrist-worn accelerometer (Activinsights, Kimbolton, UK) for 7 consecutive days during each trimester (gestational weeks: 8–13, 18–22 and 26–31 weeks). Devices will be initialised by research nurses and worn on the non-dominant wrist. Data will be processed in R (R, Auckland, New Zealand) using the GGIR package^39^ to derive key metrics including daily MVPA, sedentary time and sleep.

Self-reported PA: alongside wearing the accelerometer, participants will complete a bespoke exercise diary (online or paper depending on participant preference). The diary includes 1 week of retrospective recall and 1 week of prospective recording, capturing the type, duration, timing and context of planned or structured exercise, as well as sleep. A combination of both recall and prospective diary elements will allow data capture of 14 days while limiting the involvement of participants to 7 days in each trimester

1. Assessment of diet

dietary intake will be captured using the Remote Food Photography Method.^40^ Participants will use their smartphones to photograph all meals and snacks over a 7-day period via the FoodView app (FoodView Pty, New South Wales, Australia). Photos will be analysed to characterise dietary intake in relation to glucose outcomes.

1. Assessment of glucose outcomes

The CGM and insulin pump data of the participants will be shared via secure online platforms they use routinely in clinical care. For women with T2D not using a CGM, a Freestyle Libre Pro sensor (Abbots Diabetes Care, California, USA) will be provided for each measurement period, and will be given guidance by their care team on how to use the sensor. Glucose metrics will be processed in Diametrics software (Diametrics, Exeter, UK)^41^, in accordance with the international consensus guidelines, to derive measures such as time in range (TIR) and glycaemic variability.

1. Assessment of mental wellbeing

At each measurement period, participants will complete the 20-item PAID questionnaire^42^ via Microsoft forms. The PAID questionnaire is a validated measure that assesses diabetes-related emotional well-being. Scores will be analysed alongside PA and glucose data to explore relationships between diabetes stress, PA and glucose control throughout pregnancy.

### Focus groups

Four online focus groups (via Zoom), each comprising ~4 participants, will be facilitated by a researcher from the University of Exeter (HMJ). Four participants per group is to ensure the conversation is manageable online and to encourage engagement from all participants. Each session will last ~90 min. Focus groups will explore the experiences of PA and exercise during pregnancy among women, including perceived benefits, challenges and changes in diabetes management when being active (topic guide available in online supplementary appendix 3). Discussion will also examine motivations, barriers and facilitators to PA, and the views of participants on what support or information would be most useful during pregnancy.

Topic guides will be informed by the Socio-Ecological Capability, Opportunity, Motivation–Behaviour (SeCOM-B) model of behaviour change.^43^ The SeCOM-B model integrates the Capability, Opportunity, Motivation–Behaviour framework with the Socio-Ecological Model, recognising that behaviours are influenced by multi-level factors spanning individual, interpersonal, organisational, community and policy contexts.^43^ This integrated framework is particularly appropriate for understanding current PA behaviours and identifying what needs to change and how, as it moves beyond individual-level factors to examine the broader socio-ecological systems that shape PA participation. By mapping the mechanisms of behaviour change (capability, opportunity and motivation) across different ecological levels, the SeCOM-B model provides insight into both the individual determinants of behaviour and the systemic factors that enable or constrain PA in women with pre-existing diabetes.

### Survey and interviews

An online survey (online supplemental appendix 4) hosted on Microsoft Forms has been developed using a mixture of open and closed questions. Questions explore current PA advice given to pregnant women with diabetes, confidence in PA promotion and perceived barriers/facilitators, availability of resources and training on PA in diabetes pregnancy care. Demographic data will also be collected on profession, years of experience and any relevant PA training received. Participants are asked to rate the closed question statements on a five-point Likert scale from ‘strongly agree’ to ‘strongly disagree’. Open questions provide an opportunity for participants to share further information.

Participants who have completed the survey have the option to take part in a follow-up semi-structured interview (topic guide available in online supplemental appendix 5). The interviews will be held online and last approximately 60 min, each will be audio-recorded and transcribed verbatim. The interview structure has been guided by the SeCOM-B model to ensure a comprehensive exploration of the experiences, knowledge and attitudes of HCPs towards PA promotion.

### Data analysis

#### Analysis of quantitative outcome data

To examine changes in MVPA across pregnancy, multi-level linear modelling (MLM) will be used as it accounts for the hierarchical structure of the data (repeated measures within participants, and participants nested within NHS sites). Pregnancy stage will be treated both as an ordinal variable (trimesters 1, 2 and 3) and a continuous variable (gestational week) to explore temporal patterns in PA with varying levels of granularity. The MLM framework allows both intercepts (baseline activity) and slopes (change over time) to vary within individuals and sites, enabling a robust exploration of intra-participant and inter-participant variability. Relationships between glucose metrics and PA (particularly in relation to reported exercise sessions) will also be explored using regression-based approaches adjusted for relevant confounding factors, including age, gestational age, diabetes type, insulin use and weight.

Changes in PAID scores across pregnancy will be analysed using multi-level linear models, mirroring the structure applied to PA outcomes (repeated measures within participants, participants nested within sites). Associations between PAID scores and both PA metrics (MVPA min/day and sedentary time) and glucose indicators (TIR, mean glucose and glycaemic variability) will be explored using mixed-effects regression models. These analyses will assess whether higher distress is associated with lower PA levels or poorer glucose outcomes, adjusting for relevant covariates age, gestational age, diabetes type, insulin use and weight.

### Diet analysis

Photos will be analysed by DietAI24 to provide data on energy intake, macronutrients and fibre.^44^ Relationship between these and glucose indicators (TIR, mean glucose and glycaemic variability) will be explored using mixed-effects regression models.

### Analysis of survey data

Descriptive statistics will be used to summarise participant demographics, professional backgrounds and responses to each survey item. Survey responses will be used to support interview findings.

### Analysis of qualitative data

Qualitative data from both the focus groups with pregnant women and interviews with HCPs will be analysed following the reflexive thematic analysis of Braun and Clarke.^45^ Transcripts will be coded inductively and deductively in NVivo (Lumivero, Massachusetts, USA) to identify themes related to lived experiences, perceived barriers and facilitators related to PA during pregnancy. A reflexive, iterative approach will allow for recognition of both individual variation and broader contextual factors. Coding will be carried out by at least two members of the research team with theme development discussed collaboratively to enhance rigour. Findings will be illustrated with anonymised quotes from participants.

### Missing data

The extent and patterns of missing data will be assessed prior to analysis. Where missing data are minimal, analyses will be conducted using available data. For key outcome variables, patterns of missingness will be explored to assess whether data are missing at random. The approach to handling missing data will be reported transparently.

### Data management

All data will be pseudonymised using unique study IDs and stored on secure, password-protected servers at the University of Exeter. Data transfer between NHS sites and the research team will occur via SharePoint (Microsoft, Washington, USA) or Secure Electronic File Transfer systems. Only authorised study personnel will have access to identifiable data, which will be stored separately from research datasets.

### Patient involvement

A public and patient involvement (PPI) group was consulted during application of funding. The PPI group felt that determining how PA levels changed across pregnancy, how exercise changes glucose levels and the facilitators and barriers to PA in pregnant women with diabetes was important. Answering these questions would allow HCPs to provide specific support and reassurance to pregnant women with diabetes. They, however, felt that if we were going to look at how PA affects glucose levels, this should be done in the ‘real world’ rather than in the laboratory as it would better reflect the patients lived experience. Therefore, we decided against exercise in a controlled laboratory setting and instead proposed a real-world observational study using linked data from accelerometers and CGMs to examine how free-living PA and exercise affect glucose.

In addition to this, a patient representative has reviewed the participant-facing documentation to ensure its clarity and acceptability.

PPI representatives will be invited to contribute to the analysis and interpretation of findings, particularly in ensuring that themes and quantitative results reflect patient priorities and lived experiences. They will also support the development of clear, accessible dissemination materials, including lay summaries, patient-facing guidance and presentations at local events. Their input will help ensure the outputs of this study are meaningful, understandable and relevant to women with pre-existing diabetes and the HCPs supporting them.

### Ethics and dissemination

The protocol for objectives 1–4 was approved by East Midlands Nottingham 1 Research Ethics Committee (25/EM/0190). The protocol for objective 5 was approved by the University of Exeter Public Health and Sport Sciences ethics committee (9978878). Both are being conducted in accordance with the Declaration of Helsinki and Good Clinical Practice. All participants will receive detailed participant information sheets describing the study procedures and risks, approved by the Research Ethics Committee (included in supplementary materials as online supplementary materials appendix 1 and appendix 2). Participants will be given time to consider participation before providing informed consent, with a copy provided for their records. Participant confidentiality will be strictly maintained: each participant will be assigned a unique study ID, and all study data will be recorded, stored and analysed in a pseudonymised form, with no directly identifying information (such as name, date of birth, full postcode, NHS number or contact details) included in the analytical dataset. The link between identifiers and study IDs will be held in a separate, encrypted file on secure NHS/university servers with access restricted to authorised study personnel only, in line with UK General Data Protection Regulation and the Data Protection Act 2018. Only aggregate, non-identifiable data will be reported in publications and presentations. Participants may withdraw from the study at any time without reason, and this will not affect their medical care.

Following study completion, data will be analysed and published in peer-reviewed journals and presented at national and international academic and healthcare conferences. In addition to this, it is our intention that the results of the study are presented in a lay-friendly manner during a dedicated event at each participating site. Both participants and HCPs will be invited to attend and learn about the findings. These presentations will be scheduled after the completion of the study.

## Supplementary material

10.1136/bmjopen-2026-118879 online supplemental file 1

10.1136/bmjopen-2026-118879 online supplemental file 2

10.1136/bmjopen-2026-118879 online supplemental file 3

10.1136/bmjopen-2026-118879 online supplemental file 4

10.1136/bmjopen-2026-118879 online supplemental file 5
