# Supplementary material for: A mixed-methods longitudinal observational study exploring physical activity during pregnancy in women with pre-existing diabetes, support needs and associations with diabetes management: a study protocol
Source: BMJ Open. 2026 Jun 10;16(6):e118879. doi: 10.1136/bmjopen-2026-118879 (PMC13264927; doi:10.1136/bmjopen-2026-118879)
Supplement: online supplemental file 1 [file bmjopen-16-6-s001.docx]

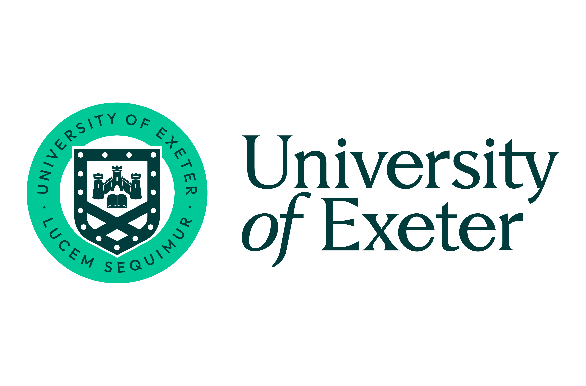


**Summary Participant Information Sheet for** Physical activity during pregnancy in women with Type 1 or Type 2 Diabetes.

**Purpose of the study**

The purpose of the study is to explore physical activity in women with diabetes during pregnancy, and how these changes may be linked to blood glucose and mental health.

The aim is to understand when support and guidance regarding physical activity might be most useful during pregnancy and to help create new guidance for health care professionals to provide this support*.*

**Who can take part?**

Women with pre-existing (Type 1 or Type 2 Diabetes) currently in their first or second trimester of pregnancy are invited to participate.

**What would taking part involve**? If you choose to take part, you will:

1. Complete a short questionnaire about yourself and your diabetes.
2. Wear a wrist-worn activity tracker for 7 days on three occasions during your pregnancy
3. Record your exercise and sleep in a study diary for 7 days on three occasions
4. Record what you eat via the FoodView mobile app for 7 days on three occasions
5. Complete a questionnaire in each trimester about your diabetes management on three occasions
6. Wear or provide glucose data from your continuous glucose monitor for 7 days on three occasions

This study will not require any changes to your routine or medication and will fit within your usual pregnancy care schedule.

**Potential risk and benefits**

There are no known large risks of participating in this study. There is a small risk of irritation from the activity tracker, if this becomes severe you can remove it. There are no direct benefits of participation, however this research will contribute to improved guidance on safe and beneficial physical activity during pregnancy.

**How will we use information about you?**

In this research study we will use information from you. We will only use information that we need for the research study. We will let very few people know your name or contact details, and only if they really need it for this study.

Everyone involved in this study will keep your data safe and secure. We will also follow all privacy rules.

At the end of the study we will save some of the data in case we need to check it and/or for future research.

We will make sure no-one can work out who you are from the reports we write.

The information pack tells you more about this.


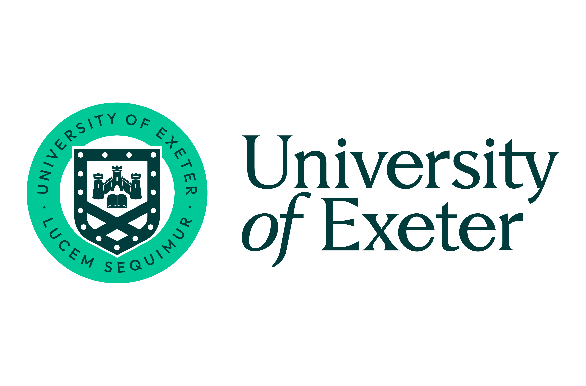


**Main Participant Information Sheet**

**Study title:** Physical activity during pregnancy in women with pre-existing diabetes, support needs, and associations with diabetes management and mental health

**Researcher name:** Dr Richard Pulsford and Holly Mei Jones

**Invitation and brief summary**

We invite you to join a research study about physical activity during pregnancy in women with Type 1 or Type 2 Diabetes. Before you decide, please read this information to understand the purpose of the study and what it involves.​

**Purpose and background to the research**

This study aims to learn how physical activity changes during pregnancy in women with diabetes and how these changes relate to blood sugar levels and mood.​

Being active during pregnancy is important for both mother and baby. It can also help diabetes management. But many pregnant women struggle to stay active, especially those with Type 1 or Type 2 Diabetes.

At the moment, health care professionals can only offer general advice about physical activity in pregnancy. Most of this advice is based on research in women without diabetes. That means it might not meet the specific needs of women with pre-existing diabetes, especially when it comes to managing blood sugar levels during exercise.

We want to better understand how physical activity changes during pregnancy in women with diabetes. We’ll also look at how it relates to blood sugar levels and mental health. To do this, we’re inviting 175 women to take part, 75 with Type 1 diabetes and 100 with Type 2 diabetes.

**What would taking part involve?**

If you decide to take part, you'll be asked to sign a consent form to show you understand what the study involves and that you're happy to participate.

Here’s what you would do:

1. Complete a short questionnaire – You'll answer some questions about yourself and your diabetes.
2. Wear an activity tracker – You’ll wear a wrist-worn monitor (like a watch) for seven days at three different points in your pregnancy. A researcher will give you the tracker at one of your regular pregnancy clinic visits.
3. Keep a study diary – You’ll note down the exercise you’ve done over the past week and log any new activity as it happens for seven days at three different points in your pregnancy. You can do this on paper or online.
4. Log your meals – You’ll take a photo before and after each meal using the FoodView mobile app for seven days at three different points in your pregnancy.
5. Complete a diabetes management questionnaire – After each seven days at three different points in your pregnancy, you’ll fill out a short questionnaire about how you’re managing your diabetes.
6. Share glucose data – If you already use a continuous glucose monitor (CGM), we’ll ask for your permission to access your data. If you don’t use one, we’ll provide one that sticks to the back of your upper arm.
7. Return the equipment – You’ll return the activity monitor and diary at your next clinic visit.

You won’t need to change your routine or medication, and you won’t need to attend any extra clinic visits.

**What are the possible benefits of taking part?**

You may not see a direct benefit from taking part, but your involvement will help us understand more about physical activity during pregnancy in women with diabetes. This could lead to better guidance and support for future patients, helping to promote healthier pregnancies.

**What are the possible disadvantages and risks of taking part?**

We have taken steps to reduce risks. Taking part in this study has minimal risks.

You might experience mild skin irritation from wearing the wrist monitor. We’ll give you advice on how to manage this. If it becomes uncomfortable, you can remove it and let us know.

One of the questionnaires includes questions about mood, which might feel sensitive. You can skip any questions or choose not to complete this part. If anything feels difficult, we can guide you to support services.

To ensure your privacy and anonymity, all personal and study data will be stored securely in accordance with University guidelines. Any identifiable information will be stored separately from study data. All study data collected will be anonymised and results will be reported ensuring you cannot be identified.

**Will I receive payment for taking part?**

You will receive a £10 voucher for each measurement completed as a thank you for your time.

**How will we use information about you?**

We will need to use information from you for this research project.

This information will include

- Activity data – how much you move during the day and night while wearing the tracker.
- Glucose data – either from your current CGM (with your permission) or from a CGM we provide.
- Questionnaire and diary data – general background information, details about your physical activity, meals, and sleep.
- Your initials and contact email address – this is so we can contact you if required.

People will use this information to do the research or to check your records to make sure that the research is being done properly.

People who do not need to know who you are will not be able to see your name or contact details. Your data will have a code number instead.

The University of Exeter the sponsor of this research.

The University of Exeter is responsible for looking after your information. We will share your information related to this research project with the following types of organisations:

- NHS trusts involved in delivering the study
- The University of Exeter research team
- Regulatory bodies (for example, research Ethics Committees, study sponsors)

We will keep all information about you safe and secure by:

- Removing any personal details from questionnaires, study diaries and CGM data.
- Using secure methods to share data within the research team so that no-one outside the study can access it.
- Storing all study data on a secure, password-protected server at University of Exeter that only the research team can access.

### **International transfers**

Your data will not be shared outside of the UK

**How will we use information about you after the study ends?**

Once we have finished the study, we will keep some of the data so we can check the results. We will write our reports in a way that no-one can work out that you took part in the study.

We will keep your study data for a maximum of 10 of years. The study data will then be fully anonymised and securely archived or destroyed.

**What are your choices about how your information is used?**

You can stop being part of the study at any time, without giving a reason, by contacting your clinical team and letting them know. If you choose to withdraw, we will keep the information that has already been collected about you.

You have the right to ask us to remove, change or delete data we hold about you for the purposes of the study. You can also object to our processing of your data. We might not always be able to do this if it means we cannot use your data to do the research. If so, we will explain why.

You will have up to two weeks after taking part to request that your data be withdrawn, as long as it has not yet been anonymised. After that point, it will no longer be possible to remove your data from the study, as it will have been anonymised and will not be identifiable.

If you agree to take part in this study, you will also have the option to allow your anonymised data to be used in future research.

**Where can you find out more about how your information is used?**

You can find out more about how we use your information:

- at [www.hra.nhs.uk/information-about-patients/](https://www.hra.nhs.uk/information-about-patients/)
- our leaflet available from: <https://www.exeter.ac.uk/about/oursite/privacy/research/#a5><http://www.hra.nhs.uk/patientdataandresearch>
- by asking one of the research team
- by sending an email to Holly Jones at hj409@exeter.ac.uk
- by sending an email to the University of Exeter’s Information Governance Manager and Data Protection Officer: [*informationgovernance@exeter.ac.uk*](mailto:informationgovernance@exeter.ac.uk), or
- by ringing us on 01392 726621

**What will happen to the results of this study?**

The results of this study will help us better understand how physical activity changes during pregnancy in women with pre-existing Type 1 and Type 2 diabetes, and how these changes may be linked to blood glucose levels and mental health.

We will share the findings through scientific journals and conferences. Your personal details will not be included in any reports, so you cannot be identified.

After the study is finished, we’ll also present the findings at an event held at your local site, where you’ll have the opportunity to hear about the results and ask questions if you’d like.

**Who has reviewed the study?**

All research in the NHS is looked at by an independent group of people, called a Research Ethics Committee, to protect your interests. This study has been reviewed and given a favourable opinion by the East Midlands - Nottingham 1 Research Ethics Committee. It has also been reviewed by the Health Research Authority (HRA) to obtain HRA approval.

This study is part of a PhD research project by Holly Mei Jones, funded by the Economic and Social Research Council through the South West Doctoral Training Partnership.

The University of Exeter is the insurer of this study.

**Contact details for further information**

If you have any questions or there is anything you wish to discuss, please contact the Chief Investigator Richard Pulsford ([R.Pulsford@exeter.ac.uk](mailto:R.Pulsford@exeter.ac.uk)).

The Sponsor for this study is the University of Exeter. The Sponsor representative is: Suzy Wignall, Senior Clinical Research Governance Manager, Research Ethics, Governance & Compliance, Research Services, University of Exeter, The Innovation Centre, Rennes Drive, Exeter, EX4 4RN. Email: [res-sponsor@exeter.ac.uk](mailto:res-sponsor@exeter.ac.uk)

If you are not happy with any aspect of the project and wish to complain please contact Richard Pulsford ([R.Pulsford@exeter.ac.uk](mailto:R.Pulsford@exeter.ac.uk)). Alternatively if you wish to contact someone outside the research team, please contact Suzy Wignall ([res-sponsor@exeter.ac.uk](mailto:res-sponsor@exeter.ac.uk)).

Thank you for your interest in the study
