# Supplementary material for: A mixed-methods longitudinal observational study exploring physical activity during pregnancy in women with pre-existing diabetes, support needs and associations with diabetes management: a study protocol
Source: BMJ Open. 2026 Jun 10;16(6):e118879. doi: 10.1136/bmjopen-2026-118879 (PMC13264927; doi:10.1136/bmjopen-2026-118879)
Supplement: online supplemental file 2 [file bmjopen-16-6-s002.docx]

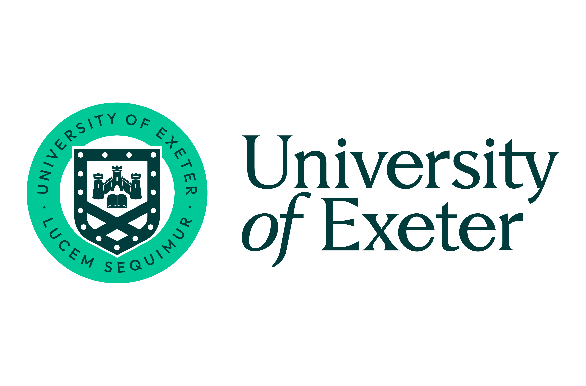


**Summary Participant Information Sheet for** Physical activity during pregnancy in women with pre-existing diabetes.

**Purpose of the study**

We want to understand what it’s like to be physically active during pregnancy when you have Type 1 or Type 2 diabetes. We’re looking at how this might affect your physical and mental health. We also want to know what makes it easier or harder to stay active, and what kind of support would help.

**Who can take part?**

Any pregnant women with Type 1 or Type 2 Diabetes at any stage in pregnancy.

**What would taking part involve?**

You’ll be invited to join a group discussion (called a focus group) with a researcher and around four other pregnant women. The group will meet online using Zoom.

We’ll talk about your experience of managing diabetes during pregnancy, especially around being active. There are no right or wrong answers, we just want to hear your thoughts.

The discussion will last about 90 minutes. You can leave the group at any time without giving a reason.

**Potential risk and benefits**

There are no major risks, but some topics might feel personal or emotional. If anything upsets you, we can suggest support services that may help.

You may not benefit directly, but your views will help improve advice and support for other pregnant women with diabetes.

**How will we use information about you?**

In this research study we will use information from you. We will only use information that we need for the research study. We will let very few people know your name or contact details, and only if they really need it for this study.

Everyone involved in this study will keep your data safe and secure. We will also follow all privacy rules.

At the end of the study we will save some of the data in case we need to check it and/or for future research.

We will make sure no-one can work out who you are from the reports we write.

The information pack tells you more about this.


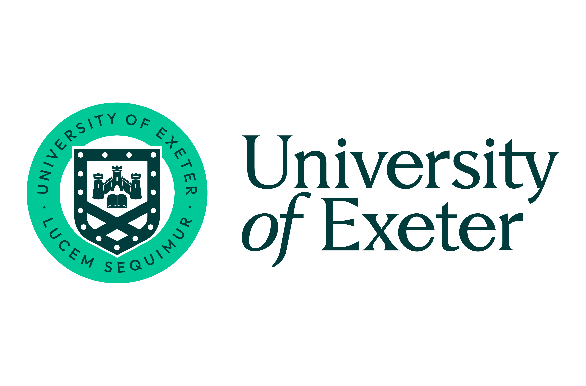


**Physical activity during pregnancy in women with pre-existing diabetes – Focus Groups**

**Study title:** Physical activity during pregnancy in women with pre-existing diabetes, support needs, and associations with diabetes management and mental health

**Researcher name: Richard Pulsford/Holly Mei Jones**

**Invitation and brief summary**

You are invited to take part in a research study. The study involves joining an online focus group to talk about your experiences with physical activity, diabetes management, and support during pregnancy.

Before you decide, it’s important to understand what the study is about and what taking part would involve. Please take time to read this information carefully. You can also talk to others about it. If you have any questions, please contact us using the details at the end.

**Purpose and background to the research**

We want to understand the experiences of pregnant women with Type 1 or Type 2 diabetes, especially around being physically active. We are interested in what helps or makes it difficult to stay active, how diabetes affects physical activity and what support, advice, or resources are helpful.

Staying active in pregnancy is linked with better physical and mental wellbeing. However, we know that many women with diabetes face extra challenges – such as managing blood glucose levels, balancing activity with fatigue, or uncertainty about what’s safe.

We’d like to learn from your experience to understand what kinds of support would make a real difference. Your insights will help us improve the guidance and resources available to pregnant women with diabetes.

For this, we are recruiting 16 women (8 each with Type 1 and Type 2 Diabetes) to take part in a group discussion.

**What would taking part involve?**

If you choose to take part, you will be invited to join a small online focus group with around three to four other pregnant women with Type 1 or Type 2 diabetes. A researcher will guide the discussion, which will last about 90 minutes (please allow up to two hours).

You’ll be asked about your experiences with physical activity during pregnancy, how diabetes has affected your ability to be active, and what support or advice has been helpful (or lacking). There are no right or wrong answers – we’re simply interested in your perspective. The session will be audio recorded so we can accurately capture what’s said.

If you prefer a more private option, or can’t attend a group session, you can choose to take part in a one-to-one interview instead. Interviews cover the same topics and can be done online or by phone, at a time that works for you.

Focus groups and interviews will be scheduled based on interest and availability. You will not need to make any changes to your usual routine, medication, or clinic visits.

**What are the possible benefits of taking part?**

You may not benefit directly, but your views will help shape better support for pregnant women with diabetes in the future.

This is an opportunity to take part in a research project and share your experiences in a meaningful way. You may find the discussion interesting and valuable, as it allows you to connect with others who have similar experiences. By taking part, your will help us gather valuable information which will be used to help improve the support pregnant women with diabetes received about physical activity.

**What are the possible disadvantages and risks of taking part?**

There are no physical risks in taking part. However, some topics may be sensitive or emotional. You can skip any question or take a break at any time.

If you do find any part of the study upsetting, we can provide information on support services that can help. Your privacy is protected, and all responses will remain anonymous

**Will I receive payment for taking part?**

If you participate in a focus group or interview, you will receive a £20 voucher as a thank you for your time. This will be arranged after your participation and will be emailed to you via the email provided to invite you to the focus group or interview.

**How will we use information about you?**

We will need to use information from you for this research project.

This information will include:

- Your initials and contact email address – this is so we can send you details of how to join the focus group.
- Focus group recordings – we will make an audio recording of the discussion.
- Written transcripts – the recordings will be typed up word-for-word, but your name and any identifying details will be removed.
- Background information – such as your age, type of diabetes, and other general information you choose to share.

Only the research team and people checking that the study is being carried out properly will see this information. Anyone who does not need to know who you are will not see your name or contact details. Instead, your data will be linked to a code number.

The University of Exeter is the sponsor of this research and is responsible for keeping your information safe. We may share your information only with the following organisations:

- NHS trusts involved in delivering the study
- The University of Exeter research team
- Regulatory bodies (for example, Research Ethics Committees and study sponsors)

The University of Exeter is the sponsor of this research and is responsible for looking after your information. We will keep all information about you safe and secure by:

- Removing names and other identifying details from transcripts as the earliest opportunity.
- When reporting findings, we will replace your name with a pseudonym, and we will remove any personal identifiers to maintain your anonymity
- Using secure methods to share anonymised data within the research team so that no one outside the study can access it.
- Storing all study data on a secure, password-protected server at the University of Exeter, which only the research team can access.

**International Transfers**

Your data will not be shared outside the UK.

**How will we use information about you after the study ends?**

Once we have finished the study, we will keep some of the data so we can check the results. We will write our reports in a way that no-one can work out that you took part in the study.

We will keep your study data for a maximum of 10 of years. The study data will then be fully anonymised and securely archived or destroyed.

**What are your choices about how your information is used?**

You can stop being part of the study at any time, without giving a reason, but we will keep information about you that we already have.

You have the right to ask us to remove, change or delete data we hold about you for the purposes of the study. You can also object to our processing of your data. We might not always be able to do this if it means we cannot use your data to do the research. If so, we will tell you why we cannot do this.

Because this study involves focus group discussions, please be aware that if you choose to withdraw, we may not be able to fully remove the comments you made during the group session, as they may be interwoven with contributions from others. However, we will do our best to identify and remove your contributions from the transcript if you withdraw within two weeks of taking part. After this point, the data will be anonymised, and it will no longer be possible to identify or remove individual contributions.

If you agree to take part in this study, you will have the option to take part in future research using your data saved from this study.

**Where can you find out more about how your information is used?**

can find out more about how we use your information:

- At [www.hra.nhs.uk/information-about-patients/](http://www.hra.nhs.uk/information-about-patients/)
- our leaflet available from: <https://www.exeter.ac.uk/about/oursite/privacy/research/#a5><http://www.hra.nhs.uk/patientdataandresearch>
- by asking one of the research team
- by sending an email to [hj409@exeter.ac.uk](mailto:hj409@exeter.ac.uk)
- by sending an email to the University of Exeter’s Information Governance Manager and Data Protection Officer: [*informationgovernance@exeter.ac.uk*](mailto:informationgovernance@exeter.ac.uk), or
- by ringing us on 01392 726621

**What will happen to the results of this study?**

We will audio record each focus group and then type up a written transcript of the discussion. To understand the key themes, we will combine all the transcripts from each group that we run and look for the common ideas and experience in what people have said.

Once we have done this, we intend to produce a short reported on the findings that we will publish in a peer-reviewed academic journal. Anonymised quotes from the focus groups may be used in these journal articles.

We can also send you a copy of the report. You can also request to be sent a link to any major findings from the project by ticking the relevant box.

**Who has reviewed the study?**

All research in the NHS is looked at by an independent group of people, called a Research Ethics Committee, to protect your interests. This study has been reviewed and given a favourable opinion by the East Midlands – Nottingham 1 *[Name of REC]* Research Ethics Committee. It has also been reviewed by the Health Research Authority (HRA) to obtain HRA approval.

This study is part of a PhD research project by Holly Mei Jones, funded by the Economic and Social Research Council through the South West Doctoral Training Partnership.

The University of Exeter is the insurer of this study.

**Contact details for further information**

If you have any questions or there is anything you wish to discuss, please contact the Chief Investigator Dr Richard Pulsford [r.pulsford@exeter.ac.uk](mailto:r.pulsford@exeter.ac.uk).

The Sponsor for this study is the University of Exeter. The Sponsor representative is: Suzy Wignall, Senior Clinical Research Governance Manager, Research Ethics, Governance & Compliance, Research Services, University of Exeter, The Innovation Centre, Rennes Drive, Exeter, EX4 4RN. Email: [res-sponsor@exeter.ac.uk](mailto:res-sponsor@exeter.ac.uk)

If you are not happy with any aspect of the project and wish to complain please contact Richard Pulsford r.pulsford@exeter.ac.uk. Alternatively, if you wish to contact someone outside the research team, please contact Suzy Wignall ([res-sponsor@exeter.ac.uk](mailto:res-sponsor@exeter.ac.uk)).

Thank you for your interest in the study
