# Supplementary material for: A mixed-methods longitudinal observational study exploring physical activity during pregnancy in women with pre-existing diabetes, support needs and associations with diabetes management: a study protocol
Source: BMJ Open. 2026 Jun 10;16(6):e118879. doi: 10.1136/bmjopen-2026-118879 (PMC13264927; doi:10.1136/bmjopen-2026-118879)
Supplement: online supplemental file 3 [file bmjopen-16-6-s003.docx]

**Physical activity during pregnancy in women with pre-existing diabetes: focus group guide**

**Introduction**

Thank you for your time and taking part in this group discussion today. My name is Holly Mei Jones, and I am a PhD student at the University of Exeter. My research is focused on understanding experiences of physical activity among pregnant women with pre-existing diabetes.

What I want to discuss today are your experiences of physical activity during pregnancy, the challenges you might faces and what would help you to be more active. We’re especially interested in how managing diabetes during pregnancy affects your decisions to be physically active and what kind of support or information you would find helpful.

- Go through information sheet
- Go through consent form. Has everyone signed?

Before we start, we have a few ground rules to ensure a respectful and productive discussion:

1. Respect the opinions of others in the group - there are no right or wrong answers, everyone’s experiences are valuable.
2. Give everyone a chance to speak – if you have spoken a lot, step back; if you haven’t spoken much, feel free to share.
3. Listen to each other and talk to each other – not to me.
4. Keep discussions confidential – please respect the privacy of others in the group, and anything that is discussed within the groups, should remain in the group. Please don’t talk about anything outside of our discussion today.

We will begin with some more general questions before moving into more specific topics. Before we start, it is important for you to know that there are no right or wrong answers. We are just exploring this topic and are really interested in hearing about your thoughts and opinions. If you don’t feel comfortable answering any questions, you don’t have to.

**THEME 1 General introduction and starting questions**

- Can everyone go round and introduce themselves and share what comes to mind when I say, ‘physical activity’?
- How does this compare to ‘exercise’? Do these words mean the same thing to you?

**THEME 2 Experiences (current activity and attitudes)**

- What sort of physical activity do you do?
- Type of physical activity e.g. walking, yoga, antenatal classes
- How often?
- Has this changed throughout pregnancy?
- Has this changed from before pregnancy?

How do you feel about physical activity at the moment?

- What do you most enjoy or not enjoy about it?
- Is your activity level different from what you would like it to be? Why?
- What benefits do you see from being physically active?
- Why do you think this?
- Where has this information come from?
- Any concerns or reasons stopping you being more active?
- Have you received any advice for being physically active during pregnancy?
- Who gave you this advice?
- How helpful was it?
- How did it make you feel about physical activity during pregnancy?

**THEME 3 Impact of pregnancy on diabetes management and physical activity**

- Have you had to change how you manage your diabetes when being active during pregnancy?
- If yes, what changes have you made?
- Has this changed throughout pregnancy?
- Did you receive specific guidance on this?
- Have you faced any challenges in managing your blood glucose during physical activity?
- Any experiences with Hyper/hypoglycaemia?
- How have you made adjustments?
- Some women may need to change their insulin dose, meal timings, or snacks.

**THEME 4 BARRIERS**

We may have already touch on some of these challenges in earlier discussions, but let’s take moment to focus specifically on what might make it harder for you to be physically active during pregnancy while managing diabetes.

What challenges do you face when considering being more active or exercising?

***Let’s start by using Menti – to share some ideas ***

| **COM-B domain** | **Questions/prompts** |
| --- | --- |
| Capability  (psychological and physical) | Do you know how to be physically active during pregnancy whilst managing your diabetes? (skills/knowledge)  Are there any pregnancy-related challenges? (fatigue, nausea) |
| Opportunity  (social and physical) | Do you have time to be active? (work, childcare, family)  Do you have access to safe spaces or facilities for activity? (Weather/safety)  Are your friends/family active? |
| Motivation | Do you feel that being physically active is important for you? (Why / Why not?)  Do you worry about being physically active? (what is it you worry about?)  How confident do you feel about being physically active? |

**THEME 5 FACILITATORS**

Now let’s take a moment to focus on what might help you to be physically active during pregnancy.

What helps you to be physically active?

* **Menti – use of word clouds? ***

| **COM-B domain** | **Questions/prompts** |
| --- | --- |
| Capability | Do you feel you understand why physical activity is beneficial for pregnancy and diabetes management?  Do you know how to be physically active during pregnancy whilst managing your diabetes? (skills/knowledge) |
| Opportunity | Do you have access to facilities/time/childcare to make activity easier?  Do you have access to safe spaces or facilities for activity?  Are there social factors that encourage you to be active? |
| Motivation | Is being physically active important for you?  What motivates you to be active? (physical/mental/enjoyment/social)  What would help you feel more confident about being active? |

**THEME 6 Support and information**

**Existing guidance**

**Let’s look at some existing resources on physical activity during pregnancy (share infographic and diabetes UK webpage, and Breakthrough T1D webpage)**

- What do you like or dislike about these examples?
- What information or support would help you feel more confident to be physically active at the moment?
- Are there specific areas you would like more guidance on?
- What format would be most useful? (written resources like leaflets, flyers, online materials or direct conversations)
- What should provide this information?

- Health care professions/exercise professionals/peer support from others

- Is there anything missing from the example we looked at?
- Can you think of a different approach that would make you feel more supported and confident to be active?

**Closing questions**

- Do you have any other thoughts or views you would like to share that we haven’t covered?
- Why did you decide to participate?
- What has it felt like to participate?
- Is it what you expected? If not, what did you expect?

N.B – The topic guide has been developed to cover the key areas of inquiry. However, as is standard in qualitative research, minor adjustments to the topic guide may be made after initial focus groups. This will allow the research team to refine questions, improve clarity, and explore emerging themes to ensure that subsequent focus groups are as effective and relevant as possible.
