# Supplementary material for: A mixed-methods longitudinal observational study exploring physical activity during pregnancy in women with pre-existing diabetes, support needs and associations with diabetes management: a study protocol
Source: BMJ Open. 2026 Jun 10;16(6):e118879. doi: 10.1136/bmjopen-2026-118879 (PMC13264927; doi:10.1136/bmjopen-2026-118879)
Supplement: online supplemental file 4 [file bmjopen-16-6-s004.docx]

University of Exeter
Electronic Survey

(hosted on MS Forms)

Title of Project: Physical activity during pregnancy in women with pre-existing diabetes - health care professionals' experiences

REC reference:

**Chief Investigator:** Holly Mei Jones (University of Exeter)

**Investigators:** Ms Holly Mei Jones, Dr Richard Pulsford, Dr Emma Cockcroft, Dr Robert Andrews

| Please read and answer the following questions | |
| --- | --- |
| **Section 1: Demographic and professional background** | |
| Question | Response |
| **Section 1: Knowledge and Attitudes**  On a scale of 1 to 5, rate the following statements (1 = strongly disagree, 2 = disagree, 3 = neither agree nor disagree, 4 = agree, 5 = strongly agree) | |
| 1. Helping women to be physically active during pregnancy is part of my role | 1-5 |
| 1. Promoting physical activity is important in my role | 1-5 |
| 1. I don’t provide physical activity advice unless specifically asked | 1-5 |
| 1. I have sufficient knowledge to advise on physical activity | 1-5 |
| 1. When discussing physical activity, I provide advice on blood glucose management | 1-5 |
| **Section 2: Factors affecting Physical Activity support**  On a scale of 1 to 5, rate the following statements (1 = strongly disagree, 5 = strongly agree) | |
| 1. I don’t have enough time to discuss physical activity | 1-5 |
| 1. There is a lack of educational opportunities on physical activity for this population | 1-5 |
| 1. I have support from my colleagues in promoting physical activity to this population | 1-5 |
| 1. Women are unlikely to be motivated to follow advice | 1-5 |
| 1. I feel confident giving advice to this population on being physically active | 1-5 |
| **Section 3: Challenges and Enablers**  Answer the following questions with as much or as little detail as you wish. | |
| 1. What challenges do you face when providing physical activity advise to women with pre-existing diabetes during pregnancy? | [Free text] |
| 1. What additional support or resources would help you feel more confident in advising physical activity to women with pre-existing diabetes during pregnancy? | [Free text] |
| **Section 4: Demographic and professional background** | |
| 1. What is your professional role?   e.g midwife, GP, diabetologist | Midwife  General Practitioner  Diabetologist  Nurse  Dietician  Other____[free text] |
| 1. How many years have you been working in this role?   [choose from] | <1 year  1-5 years  6-10 years  11+ years |
| 1. Do you currently provide care to pregnant women with pre-existing diabetes? | Yes  No |
| 1. Have you received any training on Physical Activity promotion for pregnant women with pre-existing diabetes?   [if yes, continues to question 17, if no, continues to question 19.] | Yes  No |
| 1. What type of training have you received? (select all that apply) | Undergraduate/postgraduate education CPD course or workshop  Online/resources/webinar  Self-directed learning  Other____ [free text] |
| 1. Did you find this training helpful? Please explain your response. | [Free text] |
| **Interview (optional)** | |
| 1. Did you provide your consent to be contacted about an interview?   [if yes, continues to question 20, if no, end of survey] | Yes  No |
| 1. Please provide your email address and we will contact you to arrange an interview at a time convenient for you. | [Free text] |
| **End of survey** | |

Thank you very much for completing this survey.
