# Supplementary material for: A mixed-methods longitudinal observational study exploring physical activity during pregnancy in women with pre-existing diabetes, support needs and associations with diabetes management: a study protocol
Source: BMJ Open. 2026 Jun 10;16(6):e118879. doi: 10.1136/bmjopen-2026-118879 (PMC13264927; doi:10.1136/bmjopen-2026-118879)
Supplement: online supplemental file 5 [file bmjopen-16-6-s005.docx]

**Physical activity during pregnancy in women with pre-existing diabetes - Health Care Professional’s experiences: Interview guide**

**Introduction**

Thank you for your time to speak to me today. My name is Holly Mei Jones, and I am a PhD student at the University of Exeter. My research is focused on physical activity during pregnancy in women with pre-existing diabetes.

In this interview, I would like to explore your experiences of support women with Type 1 or Type 2 Diabetes in being physically active during pregnancy. I am also interested in what support or resources would help you in your practice.

Before we start, I’d like to go over some key points

1. Your participation is voluntary, you do not have to answer any questions you’re uncomfortable with.
2. Everything you share will remain confidential, and any identifying details will be removed from the research.
3. This interview will last approximately 1 hour and will be audio-recorded for accuracy.

Do you have any questions before we start?

**THEME 1 General introduction and starting questions**

- Can you start by telling me a bit about your role and your experience working with pregnant women with Type 1 or Type 2 diabetes?

**Theme 2 Current Practice**

- Do you have conversations about physical activity in your practice?
- How do these conversations normally start?
- What do you currently advise women?
- Are there any specific concerns that women commonly raise with you?

**THEME 3 Barriers and Facilitators**

Let’s talk about the different things that affect how you support physical activity in pregnant women with pre-existing diabetes. This might include what helps, what gets in the way, or anything else that shapes how you approach this part of care.

| **COM-B domain** | **Questions/prompts** |
| --- | --- |
| Capability | Do you know what to advise regarding physical activity to pregnant women with pre-existing diabetes?  Are there any specific challenges in providing PA guidance to these women? (e.g. guidelines, safety concerns)  Have you received any training on PA promotion? (Any for pregnancy and diabetes?) |
| Opportunity | Do you have enough time during consultations to discuss Physical Activity? How is time prioritised in consultations?  Do you have access to resources to support physical activity promotion?  Are there local services (e.g. exercise programmes) you can refer patients to?  Are there colleagues or professional networks that support your efforts to promote PA? |
| Motivation | Do you believe that physical activity is an important aspect of diabetes management during pregnancy? Why or why not?  What motivates you to discuss PA with this group?  How confident do you feel in advising pregnant women with diabetes about PA.  Do you have any concerns about promoting PA to this group? |

**Hypos?**

**THEME 4 Support and Information**

- What information or support would help you feel more confident in promoting physical activity to pregnant women with pre-existing diabetes?
- Any specific topics? (e.g. safety considerations, exercise examples)
- What format of information would be most useful to you?
- Resources (infographic, flyer, online guidelines) or direct training (CPD – workshops)?
- How detailed should the information be? (summary or in-depth guidance)
- Who should be the key source of this information?
- Health care professionals, peers, fitness experts, a combination?

**Closing questions**

- Do you have any other thoughts or views you would like to share that we haven’t covered?
- Why did you decide to participate?
- What has it felt like to participate?
- Is it what you expected? If not, what did you expect?
