# Supplementary material for: A mixed-methods longitudinal observational study exploring physical activity during pregnancy in women with pre-existing diabetes, support needs and associations with diabetes management: a study protocol
Source: BMJ Open. 2026 Jun 10;16(6):e118879. doi: 10.1136/bmjopen-2026-118879 (PMC13264927; doi:10.1136/bmjopen-2026-118879)
Supplement: Supplementary file 6 [file bmjopen-2026-118879.reviewer_comments.pdf]

PEER REVIEW HISTORY

BMJ Open publishes all reviews undertaken for accepted manuscripts. Reviewers are asked to complete a checklist review form and are provided with free text boxes to elaborate on their assessment. These free text comments are reproduced below.

ARTICLE DETAILS

Title (Provisional)

A mixed-methods longitudinal observational study exploring physical activity during pregnancy in women with pre-existing diabetes, support needs, and associations with diabetes management: a study protocol

Authors

Jones, Holly Mei; Andrews, Robert; Cockcroft, Emma; Doeuk, Isy; Pulsford, Richard

---

VERSION 1 - REVIEW

**Reviewer**	**1**
**Name**	**Ching, Siew Mooi**
**Affiliation**	**Universiti Putra Malaysia, Family Medicine**
**Date**	**23-Mar-2026**
**COI**	**None**

---

1. Title: should include study design

2. Abstract:

Method: suggest to describe it according to different phase (study design, other detail and sample size in each phase)

3. Strength and limitation

The limitation regarding recruitment bias (Southwest region, specific ethnic groups) is acknowledged. Suggest adding any planned strategies to mitigate this bias during recruitment or analysis.

4. Eligibility: Women with gestational diabetes (GDM) are excluded but the rationale for excluding GDM is not stated in the protocol. Suggest to add a sentence justifying exclusion of GDM Sal

5. Sample size calculation pg 9 line 44-57:

Acknowledge that the PA change magnitude in the diabetes population is unknown and that sample size calculation was based on healthy populations (reference 34-35).

Describe how data adequacy will be determined (e.g., ongoing analysis during data collection, stopping when no new themes emerge).

6. Data recruitment procedure :

Exercise diary: The diary includes “one week of retrospective recall and one week of prospective recording.” This dual approach is unusual and may introduce recall bias for the retrospective component. The rationale for this design is not explained.

CGM for T2D: Women with T2D not using CGM will be provided with a Freestyle Libre Pro sensor. This is appropriate, but the protocol does not specify whether these women will receive training on sensor use or what support is available for sensor issues.

7. Focus group:

The protocol states “four online focus groups… each comprising of ~four participants.” Four participants per group is smaller than typical (6-10). Online groups may require smaller numbers, but the rationale should be stated.

Participants are recruited from the observational cohort. The protocol does not describe how the sub-sample will be selected (e.g., purposive sampling by diabetes type, PA level, or other characteristics).

8. Data analysis

Suggest to describe planned methods for handling missing data (e.g., multiple imputation, sensitivity analyses).

9. Transparency issue: The HCP survey and interview topic guides are not included.

Suggestion: Include the HCP survey questions and interview topic guides as supplementary appendices

10 -suggest to mention whether the protocol adhere to spirit checklist or not and suggest to add the confidentiality issue as well.

VERSION 1 - AUTHOR RESPONSE

Reviewer: 1
Dr. Siew Mooi Ching, Universiti Putra Malaysia, Universiti Putra malaysia

Comments to the Author:
1. Title: should include study design

Response: *Thank you for this helpful suggestion. The study design has now been incorporated into the title* ***(A mixed-methods longitudinal observational study exploring physical activity during pregnancy in women with pre-existing diabetes, support needs, and associations with diabetes management: a study protocol),*** *in accordance with journal guidance.*

2. Abstract:
Method: suggest to describe it according to different phase (study design, other detail and sample size in each phase)

Response: *Thank you for this constructive comment. The methods section of the abstract has been restructured to describe each study phase separately, including the study design and planned sample size for each component (page 2).*

3. Strength and limitation

The limitation regarding recruitment bias (Southwest region, specific ethnic groups) is acknowledged. Suggest adding any planned strategies to mitigate this bias during recruitment or analysis.

Response*: We appreciate this suggestion. Additional detail has been added to the recruitment plan outlining strategies to mitigate potential recruitment bias. These include minimising barriers to participation through remote data collection and flexible scheduling, ongoing monitoring of participant demographics, and consideration of targeted recruitment or prioritisation of under-represented groups where feasible to enhance sample diversity and representativeness.*

Added: Recruitment may be skewed towards the South West region, specific ethnic groups and those engaged with care or an interest in PA. To mitigate this, we will recruit across multiple NHS sites, including PICs to widen geographic reach, which serve socioeconomically and ethnically diverse communities. In addition to this, we will closely monitor recruitment demographics throughout the study to ensure the inclusion of individuals from ethnic minority backgrounds. (page 8)

4. Eligibility: Women with gestational diabetes (GDM) are excluded but the rationale for excluding GDM is not stated in the protocol. Suggest to add a sentence justifying exclusion of GDM.

Response: *Thank you for noting this. A sentence has been added to justify the exclusion of gestational diabetes.*

Added: Rationale: women with gestational diabetes are excluded as it arises during pregnancy (typically diagnosed during late second trimester) and has differing clinical management from T1D and T2D. *(page 10)*.

5. Sample size calculation pg 9 line 44-57:

Acknowledge that the PA change magnitude in the diabetes population is unknown and that sample size calculation was based on healthy populations (reference 34-35).

Response*: We have clarified that the expected magnitude of physical activity change in women with pre-existing diabetes is currently unknown. Therefore, the sample size calculation is informed by evidence from studies in healthy and obese pregnant populations.*

Added: The magnitude of change in PA in women with pre-existing diabetes during pregnancy is unknown, therefore the sample size has been calculated based on healthy and obese pregnant populations. (page 8)

Describe how data adequacy will be determined (e.g., ongoing analysis during data collection, stopping when no new themes emerge).

Response*: We have expanded the qualitative methods section to explain how data adequacy will be monitored through concurrent data collection and analysis, with recruitment continuing until no new themes emerge.*

Added: Data adequacy will be assessed through ongoing, iterative analysis during data collection. Recruitment will continue until the research team determines that additional focus groups or interviews are no longer generating new, substantively meaningful insights, and that the data addresses the research questions. (page 9)

6. Data recruitment procedure :
Exercise diary: The diary includes "one week of retrospective recall and one week of prospective recording." This dual approach is unusual and may introduce recall bias for the retrospective component. The rationale for this design is not explained.

Response: *Thank you for this insightful comment. We have expanded the rationale for the exercise diary in the study design. Recall of physical activity over 3 days has been validated in adults and children. This method of recall and prospective recording of exercise is being used in a study in children (GlucoseGo), involving a 2 week recall and 2 week prospective recording.*

Added: A combination of both recall and prospective diary elements will allow data capture of 14 days while limiting the participants involvement to 7 days each trimester (page 12).

CGM for T2D: Women with T2D not using CGM will be provided with a Freestyle Libre Pro sensor. This is appropriate, but the protocol does not specify whether

these women will receive training on sensor use or what support is available for sensor issues.

Response: *Thank you for raising this point. Additional detail has now been included. Within the UK, it is standard practice that women with T2D received CGMs. If they don't have one, the team will provide training as they normally would on CGM use.*

Added: For women with T2D not using a CGM, a Freestyle Libre Pro sensor will be provided for each measurement period, and will be given guidance from their care team on how to use the sensor (page 12).

7. Focus group:
The protocol states "four online focus groups… each comprising of ~four participants." Four participants per group is smaller than typical (6-10). Online groups may require smaller numbers, but the rationale should be stated.

Response: *Thank you for this feedback, we have added a sentence to justify this (page 13).*

Added: Four participants per group is to ensure the conversation is manageable online and to encourage engagement from all participants. (page 13)

Participants are recruited from the observational cohort. The protocol does not describe how the sub-sample will be selected (e.g., purposive sampling by diabetes type, PA level, or other characteristics).

Response*: We have clarified that qualitative participants will be recruited using* purposive sampling framework.

Added: Participants for the focus groups will be recruited from the observational cohort using a purposive sampling framework. While participation in the qualitative component is voluntary, sampling will be guided by predefined characteristics to support diversity in ethnicity and socioeconomic status. Characteristics of interested participants will be monitored, and invitations will be issued to ensure representation across key demographic groups where feasible. Page 9.

8. Data analysis
Suggest to describe planned methods for handling missing data (e.g., multiple imputation, sensitivity analyses).

Response: *Thank you for this helpful suggestion. We have expanded the data analysis section to describe the planned approach to handling missing data.*

Added: Missing Data

The extent and patterns of missing data will be assessed prior to analysis. Where missing data are minimal, analyses will be conducted using available data. For key outcome variables, patterns of missingness will be explored to assess whether data are missing at random. The approach to handling missing data will be reported transparently. Page 15

9. Transparency issue: The HCP survey and interview topic guides are not included. Suggestion: Include the HCP survey questions and interview topic guides as supplementary appendices

Response: *Thank you for highlighting this. Both the healthcare professional survey, interview topic guide and focus group topic guides have now been added as supplementary appendices.*

10 -suggest to mention whether the protocol adhere to spirit checklist or not and suggest to add the confidentiality issue as well.

Response: *We have clarified that reporting will adhere to STROBE guidelines for observational analyses and COREQ guidelines for qualitative findings.*

Added: This observational study will be reported in line with the STROBE checklist, specific to observational studies [38], and the qualitative studies will be reported in line with the COREQ checklist, specific to qualitative studies [39]. (page 11)

Response: *Additional detail has been included regarding participant confidentiality and data protection procedures,*

Added: Participant confidentiality will be strictly maintained: each participant will be assigned a unique study ID, and all study data will be recorded, stored and analysed in a pseudonymised form, with no directly identifying information (such as name, date of birth, full postcode, NHS number, or contact details) included in the analytical dataset. The link between identifiers and study IDs will be held in a separate, encrypted file on secure NHS/university servers with access restricted to authorised study personnel only, in line with UK GDPR and the Data Protection Act 2018. Only aggregate, non-identifiable data will be reported in publications and presentation. (page 16)
